# Genome-wide analysis and expression profiling of the heat shock transcription factor gene family in Physic Nut (*Jatropha curcas* L.)

**DOI:** 10.7717/peerj.8467

**Published:** 2020-02-05

**Authors:** Lin Zhang, Wei Chen, Ben Shi

**Affiliations:** School of Environmental Engineering and Chemistry, Luoyang Institute of Science and Technology, Luoyang, Henan, China

**Keywords:** *Jatropha*, Heat shock transcription factor, Gene family, Abiotic stress, Gene expression

## Abstract

The heat shock transcription factor (Hsf) family, identified as one of the important gene families, participates in plant development process and some stress response. So far, there have been no reports on the research of the *Hsf* transcription factors in physic nut. In this study, seventeen putative *Hsf* genes identified from physic nut genome. Phylogenetic analysis manifested these genes classified into three groups: A, B and C. Chromosomal location showed that they distributed eight out of eleven linkage groups. Expression profiling indicated that fourteen *JcHsf* genes highly expressed in different tissues except *JcHsf1*,* JcHsf6* and *JcHsf13*. In addition, induction of six and twelve *JcHsf* genes noted against salt stress and drought stress, respectively, which demonstrated that the *JcHsf* genes are involved in abiotic stress responses. Our results contribute to a better understanding of the *JcHsf* gene family and further study of its function.

## Introduction

The heat shock transcription factor (Hsf) as the direct transcriptional activator of genes plays an important role in regulating plant growth, development and in response to abiotic stresses, such as heat, drought and salt stress (Von Koskull-Döring, Scharf & Nover, 2007). Under heat stress condition, Hsfs functioned as the molecular chaperone protects cells structure in protein folding and assembly process (Hu, Hu & Han, 2009).

Hsfs well-known as a group of DNA-binding proteins, specially recognize the binding motifs AGAAnnTTCT which conserved in the promoter regions of Hsf-inducible genes (Guo et al., 2016). Despite a considerable variety in size and sequence, the main domains of Hsf in eukaryotes are conservative. A typical Hsf was composed of the DNA binding domain (DBD), the oligomerization domain (OD), nuclear localization signal (NLS), nuclear export signal (NES), repressor domain (RD) and C-terminal activator peptide motif (AHA) (Nover et al., 2001; Guo et al., 2016). Since the first *Hsf* gene was cloned and characterized in the yeast (Wiederrecht, Seto & Parker, 1988), more and more Hsf proteins had been cloned and characterized in plants. The plants Hsfs were separated into three groups (A, B and C), and further divided into 16 subgroups (A group: A1-A9; B group: B1-B5; C group: C1-C2) (Nover et al., 2001; Hu, Hu & Han, 2009; Scharf et al., 2012). Both AHA and adjacent NES motifs in the C-terminal are specific of Hsf A, but absent in Hsf B and Hsf C (Nover et al., 2001). Increasing research indicated that HSF is involved in plant development, biotic and abiotic stress. For example, overexpression of *HsfA1a, HsfA1b, HsfA2* or *Hsf3* in *Arabidopsis* promoted its stress tolerance (Qian et al., 2014; Albihlal et al., 2018; Ogawa, Yamaguchi & Nishiuchi, 2007; Prandl et al., 1998). Similar stress tolerance phenotypes were also found in tobacco (Personat et al., 2014). In contrast, the *hsfa1a/b/d/e* quadruple mutant exhibited a severe growth retardation phenotype (Yoshida et al., 2011). Additionally, AtHsfA6a-overexpressing *Arabidopsis* demonstrate a significantly decreased germination under ABA treatment (Hwang et al., 2014). It is worth noting that *HsfA2*, which shared similar structure with *HsfA1* but with a different expression profile*,* was strongly induced under long-term or cycled heat stress conditions in *Arabidopsis* (Charng et al., 2007; Nishizawa-Yokoi et al., 2009). This indicated that even the orthologous genes play a divergent role in different plants.

Based on released genome sequences, genome-wide analyses of Hsf family performed in various plant species, such as *Arabidopsis* (Guo et al., 2008), rice (Guo et al., 2008; Mittal et al., 2009; Hu, Hu & Han, 2009), pepper (Guo et al., 2015), Chinese cabbage (Huang et al., 2015), maize (Lin et al., 2011), *Triticum aestivum* (Xue et al., 2014), tea plant (*Camellia sinensis*) (Liu et al., 2016), soybean (*Glycine max*) (Chung, Kim & Lee, 2013), *Brassica oleracea* (Lohani et al., 2019), *Salix suchowensis* (Zhang et al., 2015a) and sesame (Dossa, Diouf & Cisse, 2016). These investigations are of great significance for the function analysis of *Hsfs* refers to plant development and in response to abiotic stresses.

Physic nut (*Jatropha curcas* L.), which is well-known as a renewable resource for biodiesel production, has a high tolerance to drought and salt stress (King et al., 2009; Divakara et al., 2010). To date, the genome sequence and expression profile of physic nut under several abiotic stresses were available (Wu et al., 2015; Zhang et al., 2014; Zhang et al., 2015b). Therefore, the Hsf family in physic nut could be characterized at the molecular level. However, no detailed study of *JcHsf* family genes performed. In this study, a total of 17 putative *JcHsf* genes were identified. The classification, phylogenetic reconstruction, chromosome distribution, gene structure and conserved motifs of the *JcHsfs* were predicted and analyzed. In addition, the expression profile of *JcHsf* genes was analyzed under normal condition and in response to salt and drought stress. Taken together, these results these results provide important information for further study of functional genes in the physic nut Hsf family and will provide insights into the functional analysis of other plants *Hsf* genes.

## Materials & Methods

### Plant materials and treatment

The inbred physic nut cultivar GZQX0401 were used in this study. The seed germination, planting conditions, stress treatment and material collection were performed as described previously (Zhang et al., 2014; Zhang et al., 2015b).

### RNA isolation and gene expression analysis

Total RNA was extracted from ∼100 mg leave samples using the modified CTAB method (Zhang et al., 2014). The RNA was then reverse transcript into cDNA by M-MLV reverse transcriptase (Promega). For qRT-PCR analysis, primers used in this study were designed by primer 6.0 (Table S1). Each reaction was performed using TaKaRa Ex Taq HS Kit according to the instructions of the manufactor. The expression levels were calculated using the 2^−ΔΔCT^method. Each PCR assay was run in duplicate for three independent biological replicates. For gene expression profiles analysis, the number of expressed tags was calculated and then normalized to TPM (number of transcripts per million tags). Heatmap of Hsf family expression in different tissues was constructed based on the number of TPMs. While the heatmap of Hsf family expression under salt and drought stress was constructed by log2 conversion of TPM values.

### Identification and characteristics of *Hsf* genes in physic nut

To identify the *Hsf* family genes of physic nut, the HSF protein sequences of in *Arabidopsis* and rice was used as query sequences to execute BLASTP search against the physic nut genome. Target gene sequences were selected with *e*-value cut-off less than 1e^−10^. The Hsf protein sequences of *Arabidopsis* and rice were downloaded from TAIR (http://www.arabidopsis.org/) and the website (http://plntfdb.bio.uni-potsdam.de/v3.0/), respectively. In addition, the HMM file built based on Hsf domain (PF00447) was used to perform HMM searches against the local protein databases of the physic nut by HMMER3 (Eddy & Pearson, 2011). In order to confirm the accuracy of identified genes, the SMART program was used to detect DBD domains and coiled-coil structures (SMART: http://smart.embl-heidelberg.de/). Those protein sequences lacking the DBD domain or a coiled-coil structure were removed. Next, Clustal X was aligned to remove redundant sequences of the confirmed JcHsf sequences (Larkin et al., 2007). Finally, the online ExPasy program (http://www.expasy.org/tools/) was applied to analysis the length, molecular weight and isoelectric point parameters of each JcHsf protein.

### Structure and motif analysis of *JcHsf* genes

The exon/intron structures of *JcHsf* genes were elucidated by Gene Structure Display Server (GSDS, http://gsds.cbi.pku.edu.cn/) (Guo et al., 2007). Conserved motifs were analyzed using the MEME Suite version 5.0.5 (http://meme-suite.org/tools/meme; Bailey et al., 2009). The parameters were set as the default value except the numbers of different motifs, which was set to 30.

### Chromosomal locations, multiple sequence alignment and phylogenetic analysis of *JcHsf* genes

All identified *JcHsf* genes were mapped on the eight out of eleven linkage group base on physic nut genome database. The maximum likelihood mapping algorithm and Kosambi mapping function are used to calculate the map distance within cM (Wu et al., 2015). The linkage map of *JcHsf* genes was mapped using the Map-Chart software package. Amino acid sequences of Hsf protein were aligned by Clustal X (version 1.83). GeneDoc was then used to manually edit the results. The phylogenetic tree was constructed among *Arabidopsis thaliana*, *Oryza sativa*, *vitis vinifera* and *Jatropha curcas* L. using the maximum likelihood method in MEGA5 with 1,000 replicates (Tamura et al., 2011). The HSF proteins from four plant species were listed in Table S2.

## Results

### Identification and chromosomal localization of *JcHsf* gene family

**Figure 1:**
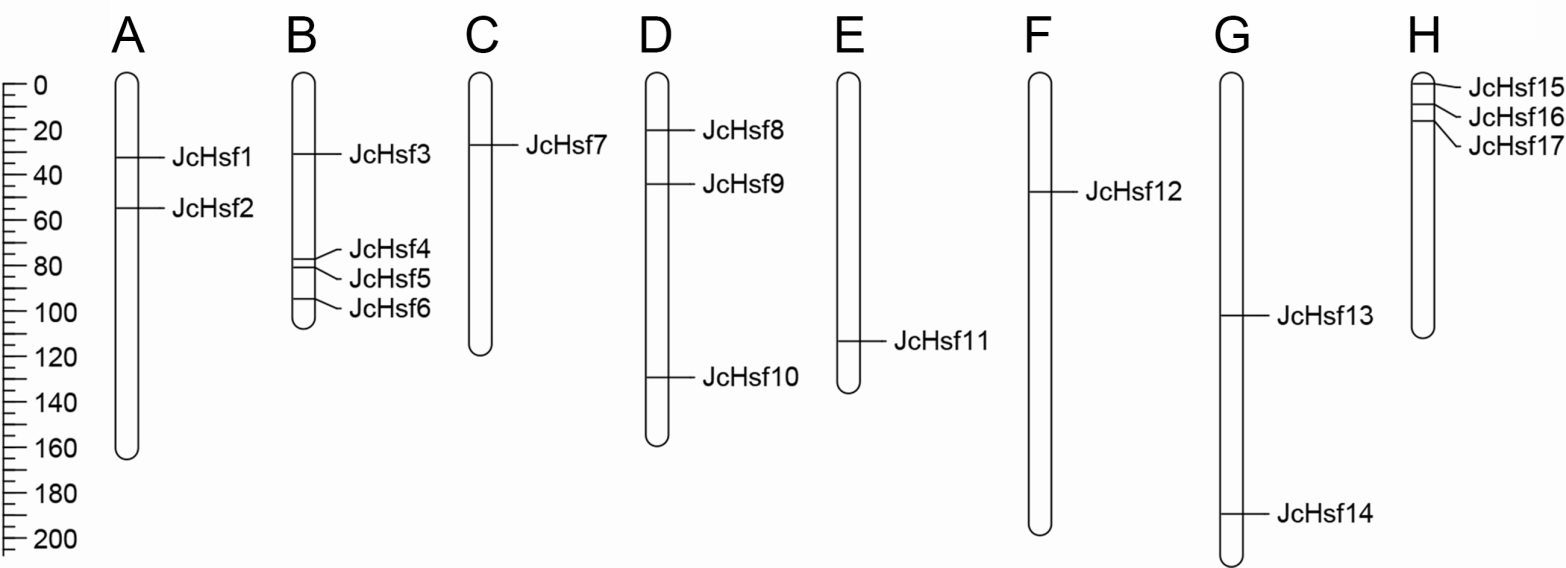
Distribution of *JcHsf* genes on physic nut chromosomes according to the linkage map. (A) Linkage group 1. (B) Linkage group 3. (C) Linkage group 5. (D) Linkage group 6. (E) Linkage group 7. (F) Linkage group 8. (G) Linkage group 9. (H) Linkage group 11. In total, 17 *JcHsf* genes were mapped to eight linkage groups (LGs). The scale is in centiMorgans (cM).

Seventeen (17) putative genes of *JcHsf* family were identified in the present study, and they were named *JcHsf1*-*17* from top to bottom according to their position on the physic nut linkage groups (LGs) 1 to 11. The accession numbers in GenBank and detailed information of *JcHsf* gene family were listed in Table 1. The JcHsf protein lengths ranged from 214 aa (JcHsf 7) to 560 aa (JcHsf 11), The molecular masses of the JcHsf proteins were predicted between 28.3 to 62.3 KDa, and the theoretical pIs were ranged from 4.67 to 9.05 (Table 1). Chromosomal location indicated that no *JcHsf* was mapped on LGs 2, 4 and 10, and 17 *JcHsf* genes were not randomly distributed in the other 8 LGs (Wu et al., 2015) (Fig. 1). Large difference in number of *JcHsf* genes located in each LG. The result showed four *JcHsf* genes on LG3, three on LG6 and 11, two on LG1 and 9, and one on LG5, 7 and 8 (Fig. 1).

**Table 1:** Accession members and characteristics of 17 *JcHsf* genes in physic nut.

Genes	Gene ID	Protein length (aa)	pI	MW (kDa)	Location
*JcHSF1*	JCGZ_02229	359	5.42	41.4	LG1
*JcHSF2*	JCGZ_01081	495	5.93	55.2	LG1
*JcHSF3*	JCGZ_17108	517	4.81	56.3	LG3
*JcHSF4*	JCGZ_04789	358	4.74	41.1	LG3
*JcHSF5*	JCGZ_21617	406	5.04	46.2	LG3
*JcHSF6*	JCGZ_21433	214	9.05	24.8	LG3
*JcHSF7*	JCGZ_26137	311	5.44	34.4	LG5
*JcHSF8*	JCGZ_21103	242	7.59	28.3	LG6
*JcHSF9*	JCGZ_06744	560	4.77	62.3	LG6
*JcHSF10*	JCGZ_02539	497	5.19	55.2	LG6
*JcHSF11*	JCGZ_12391	288	5.54	31.2	LG7
*JcHSF12*	JCGZ_21870	357	5.15	40.8	LG8
*JcHSF13*	JCGZ_03936	425	4.91	48.5	LG9
*JcHSF14*	JCGZ_20639	333	5.45	36.6	LG9
*JcHSF15*	JCGZ_07238	320	5.94	35.7	LG11
*JcHSF16*	JCGZ_07430	473	4.85	52.5	LG11
*JcHSF17*	JCGZ_07655	380	4.67	42.7	LG11

### Phylogenetic and structures analysis of *JcHsf* gene family

**Figure 2:**
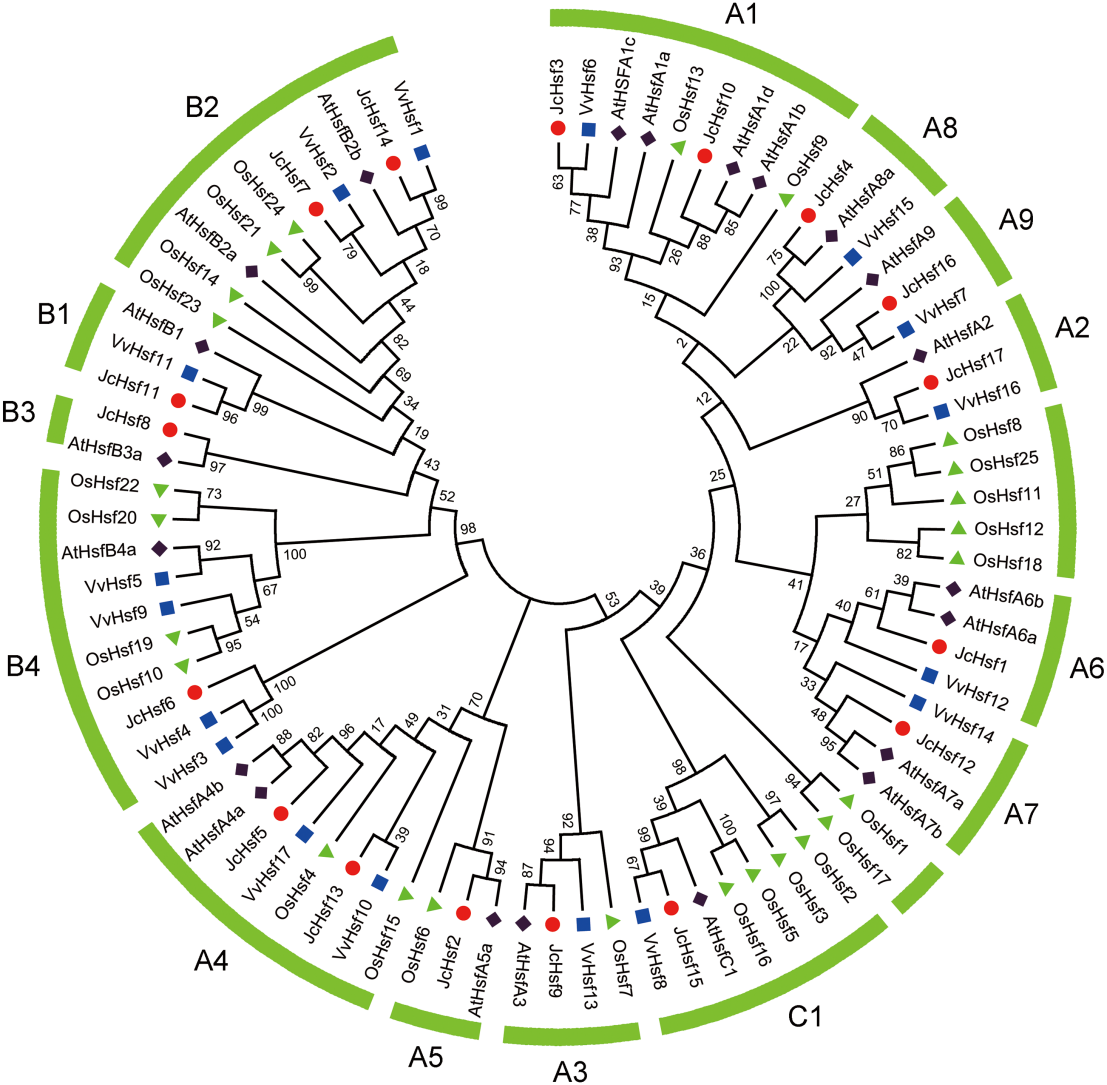
Phylogenetic relationships of *Arabidopsis*, rice, grape and physic nut Hsf proteins. The unrooted tree was constructed, using the MEGA5.0 program, by the neighbor-joining method. Bootstrap values were calculated for 1,000 replicates.

To survey the evolutionary relationships of *JcHsf* gene family, protein sequences from other three well-studied and representative species, including a dicot *Arabidopsis*, a monocot rice and grape, were selected to construct a phylogenetic tree (Fig. 2). Similar to the Hsfs from other three plant species, JcHsfs were divided into three groups (Group A, B and C) according to previous study on AtHsfs (Guo et al., 2008). The largest group A was then classified into nine subgroups (A1-A9), including 11 members (JcHsf1, JcHsf2, JcHsf3, JcHsf4, JcHsf5, JcHsf9, JcHsf10, JcHsf12, JcHsf13, JcHsf16 and JcHsf17), accounted for 64.7% of total JcHsfs. The next group B was classified into four subgroups (B1-B4), with five members (JcHsf6, JcHsf7, JcHsf8, JcHsf11 and JcHsf14), accounted for 29.4%. Only JcHsf15 was classified into the smallest group C, which represented 5.9%. Results showed a close evolutionary relationship with dicotyledons plants.

**Figure 3:**
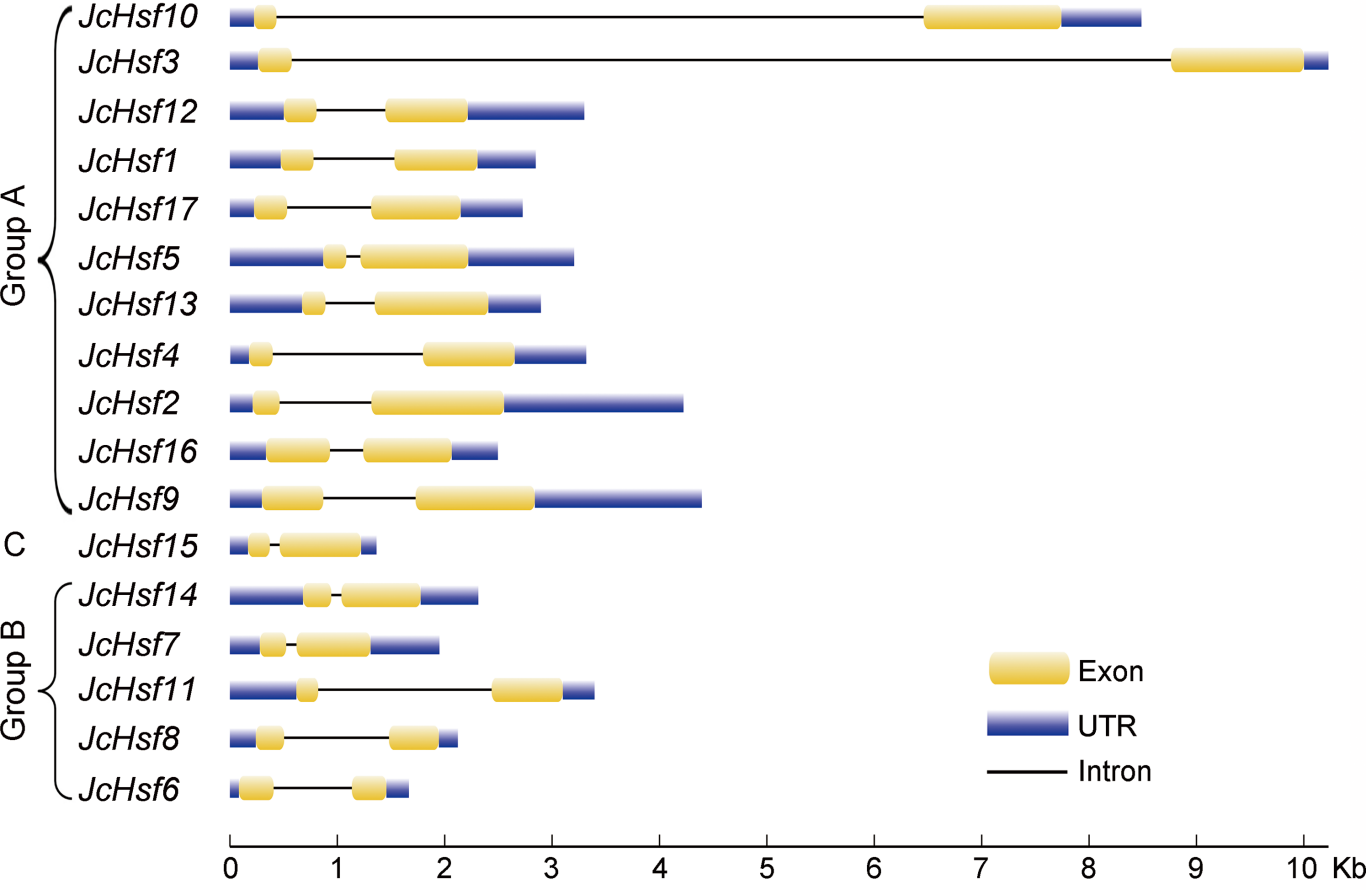
Intron-exon structure of *JcHsf* genes. Yellow boxes indicate the exon regions, black lines indicate introns and blue boxes indicate upstream and downstream UTR regions. The lengths of the boxes and lines were scaled according to the length of the genes.

During the evolution process, the exon/intron structural within a gene family appears divergence, which of great significance to study the evolution of gene families. So the exon/intron structures of *JcHsf* genes were analyzed in the present study. It was shown that all *JcHsfs* had only one intron, illustrating a very highly conserved exon/intron splicing arrangement exists in physic nut (Fig. 3). Although physic nut *Hsf* genes shared same intron number, the length of intron was different in the groups. For instance, in the subgroup A1, the intron length of *JcHsf3* was much longer than that of *JcHsf10*.

### Conserved domains and motifs of JcHSF proteins

**Figure 4:**
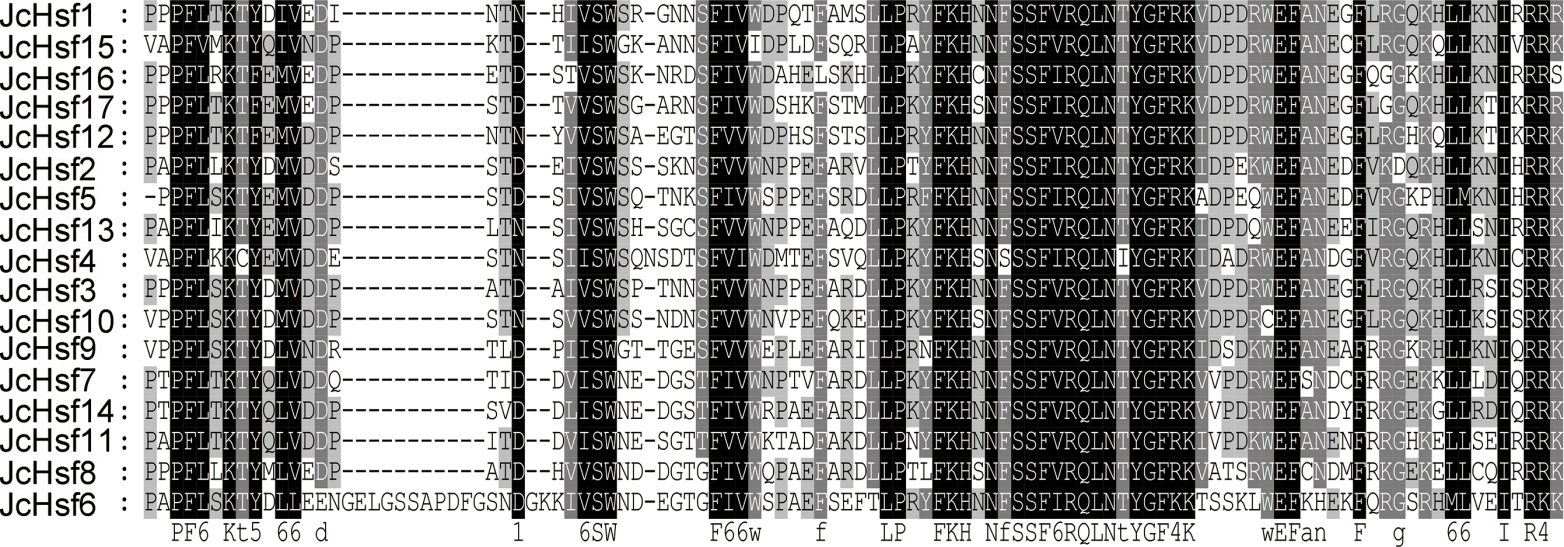
Multiple sequence alignment of the DBD domains of 17 members of the JcHsf protein family. Multiple alignment results clearly revealed highly conserved DBD domains among physic nut *Hsf* genes.

**Figure 5:**
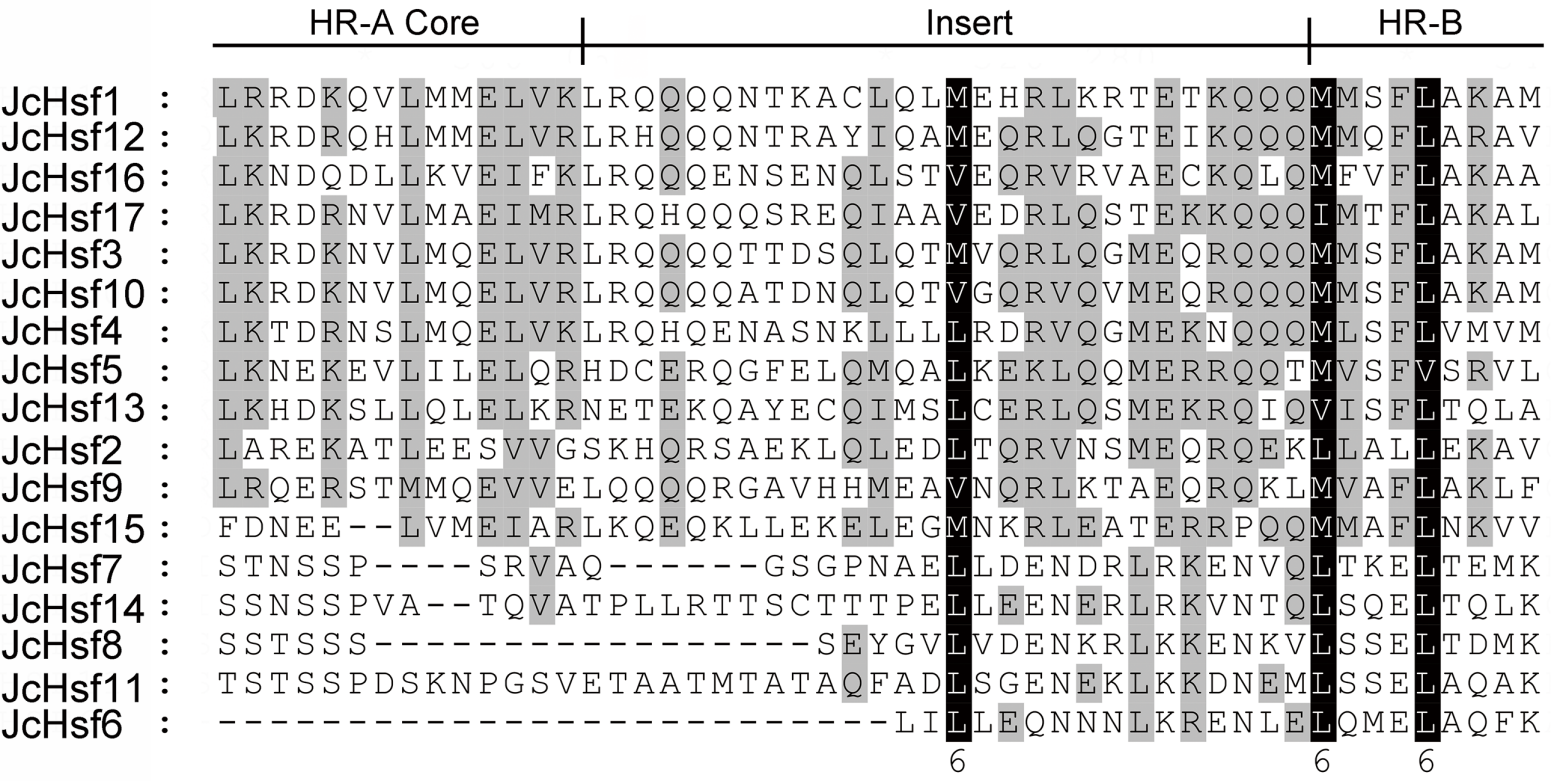
Multiple sequence alignment of the HR-A/B regions of the Hsf protein family in physic nut. The scheme at the top depicts the locations and boundaries of the HR-A core, insert, and HR-B regions within the HRA/B regions.

To well investigate the gene structure in the aspects of functional divergence, the conserved motifs/domains of the JcHsfs were analyzed based on the reported information about AtHsfs and OsHsfs (Guo et al., 2008), which contribute to discern a serious about putative functional domains for all JcHsfs. DBD, HR-A/B, NLS, NES, AHA, and RD conserved domains were confirmed in JcHsf proteins (Table 2). Multiple alignment analyses clearly showed that the DBD, most conserved section of JcHsfs, was located in the N-terminal region (Fig. 4). Based on the differences between the HR-A and HR-B regions, three types of Hsfs were identified in the physic nut (Fig. 5).

**Table 2:** Functional domains of physic nut *Hsf* genes.

Gene name	Group	DBD	NLS	NES	AHA	RD
*JcHsf1*	A6	41-134	(226)KDKMK7KKRRR	(347)LVEQLGYL	(319)EGFWDDLLNE	
*JcHsf2*	A5	23-116	(216)KKRR	(361)LNLTL	(443)DVFWEQFLTE	
*JcHsf3*	A1	44-137	(242)NRR5KKRRLK	(503)LTEKMGLL		
*JcHsf4*	A8	12-106	(205)KENNWR		(298)DSAWEQFLL	
*JcHsf5*	A4	11-104	(206)RKRR	(162)MQALKE	(342)DVFWEQFL	
*JcHsf9*	A3	130-223	(324) RMNRKFVK			
*JcHsf10*	A1	9-102	(204)KR6KKRR	(483)LTEQMELL	(432)DVFWEQFL	
*JcHsf12*	A7	41-134	(227)KDKRK7KKRRR	(345)LAERLGYL	(322)DGFWDELLSE	
*JcHsf13*	A4	12-105	(203)NKKRR	(161)IMSLCE	(365)DHFWEYFLTE	
*JcHsf16*	A9	138-231	(320)KRKRQR8KKRR	(447)LELEDL		
*JcHsf17*	A2	42-135	(230)KK9KRR	(369)LVDQMGYL	(282)ETLFSAALDD	
*JcHsf7*	B2	21-114	(257)KRGR			(249)LFGV
*JcHsf8*	B3	27-120	(179)KRKCK	(207)PKLFGVRL		(209) LFGV
*JcHsf11*	B1	7-100	(264)KTKKRGR	(254)FKLFGVLL		(256) LFGV
*JcHsf14*	B2	27-120	(282)KRMRK			(274) LFGV
*JcHsf6*	B4	34-140	(208)NKIRRL			
*JcHsf15*	C1	7-100	(184)KKRR			

**Figure 6:**
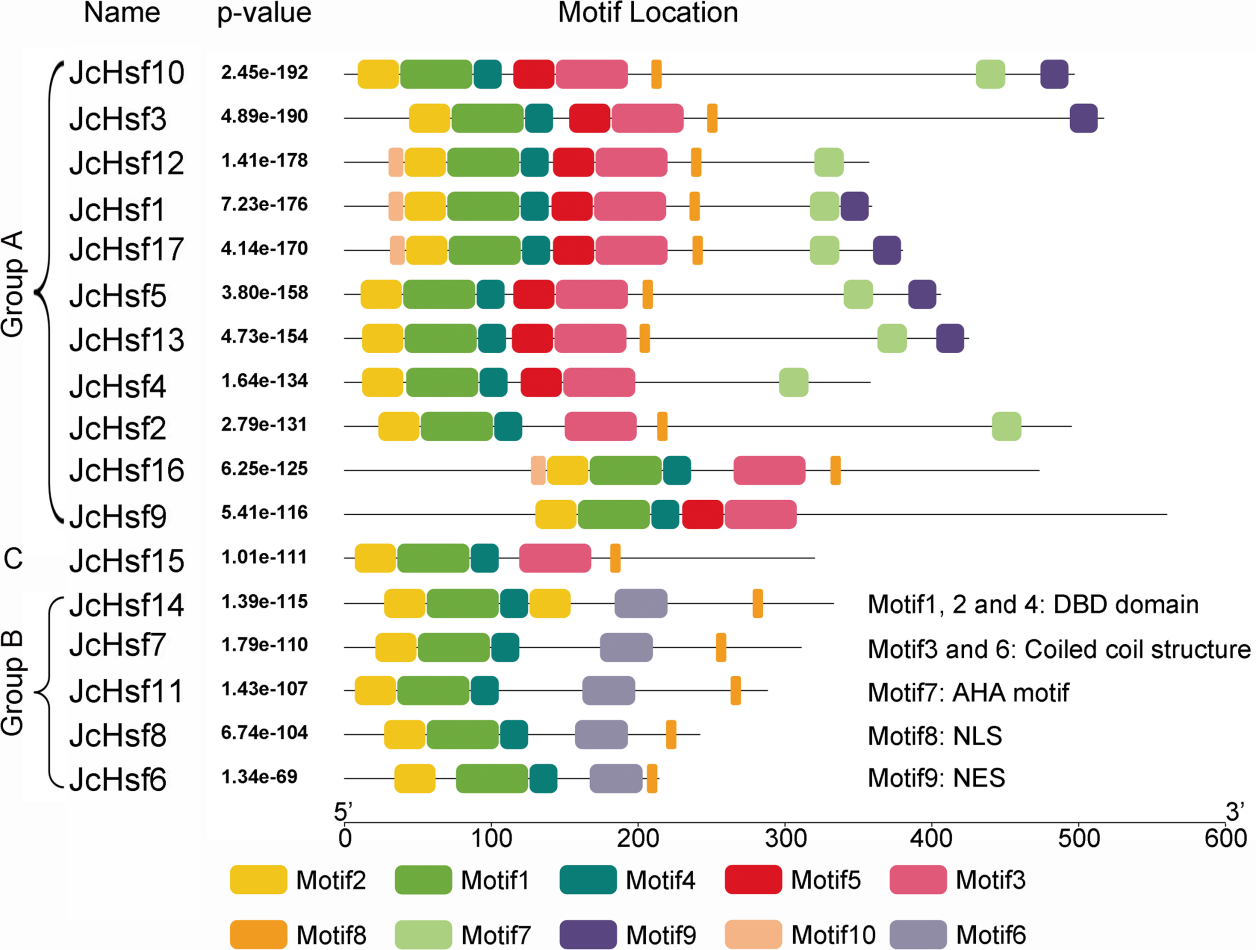
Distribution of conserved motifs in the JcHsf family members. The names of all members of the gene clusters defined and the combined *P* values are shown on the left side of the figure, while motif sizes are indicated at the bottom of the figure. Different motifs are indicated by different color numbered 1–10. For details of motifs, refer to Table S3.

**Figure 7:**
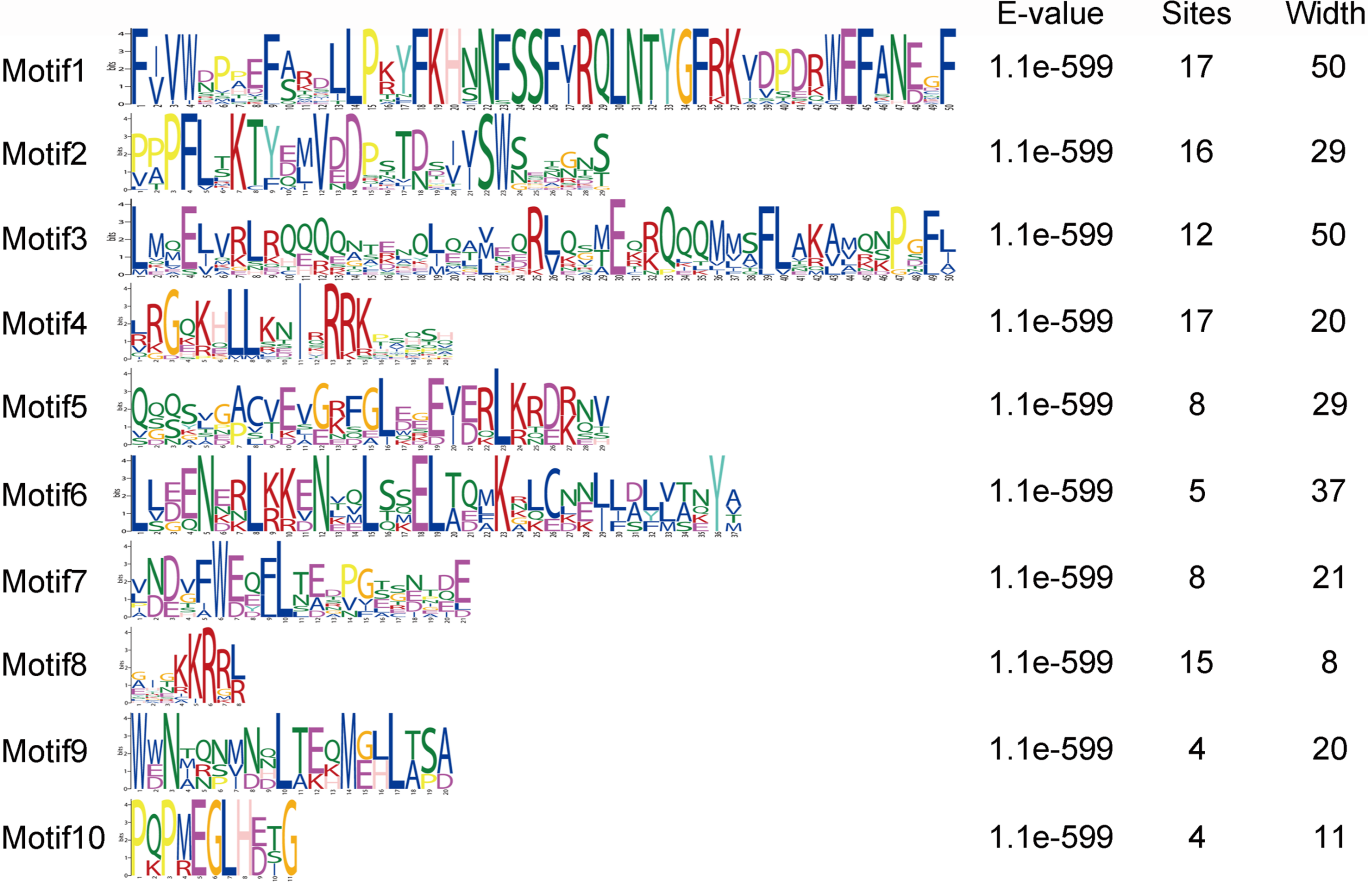
Motif logo, the amino acid composition of each conserved motif. Amino acid sequences of each motif identified by MEME tools. The font size indicates the frequency of the corresponding amino acid. Motif location and combined *E*-value are shown.

The motif of JcHsf protein was also analysis using the MEME motif search tool (Fig. 6). Among the ten detected motifs, motifs 1, 2, and 4 including the highly conserved DBD existed in all the JcHsfs (Fig. 7). The motif 6 representing the HR-A/B region was found in all class B JcHsfs, while which was replaced by motif 3 in classes A and C. Most JcHsf proteins contain Motifs 8 representing NLS except JcHsf4 and JcHsf9. Six proteins in the subgroup A1, A2, A4 and A6 had motif nine which consisted of NES. In additionally, motifs 10 containing AHA were discovered in the C-terminus of most groups A JcHsfs. Moreover, some unknown motifs were also detected in this study.

### Expression profiles of *JcHsf* genes in different tissues

**Figure 8:**
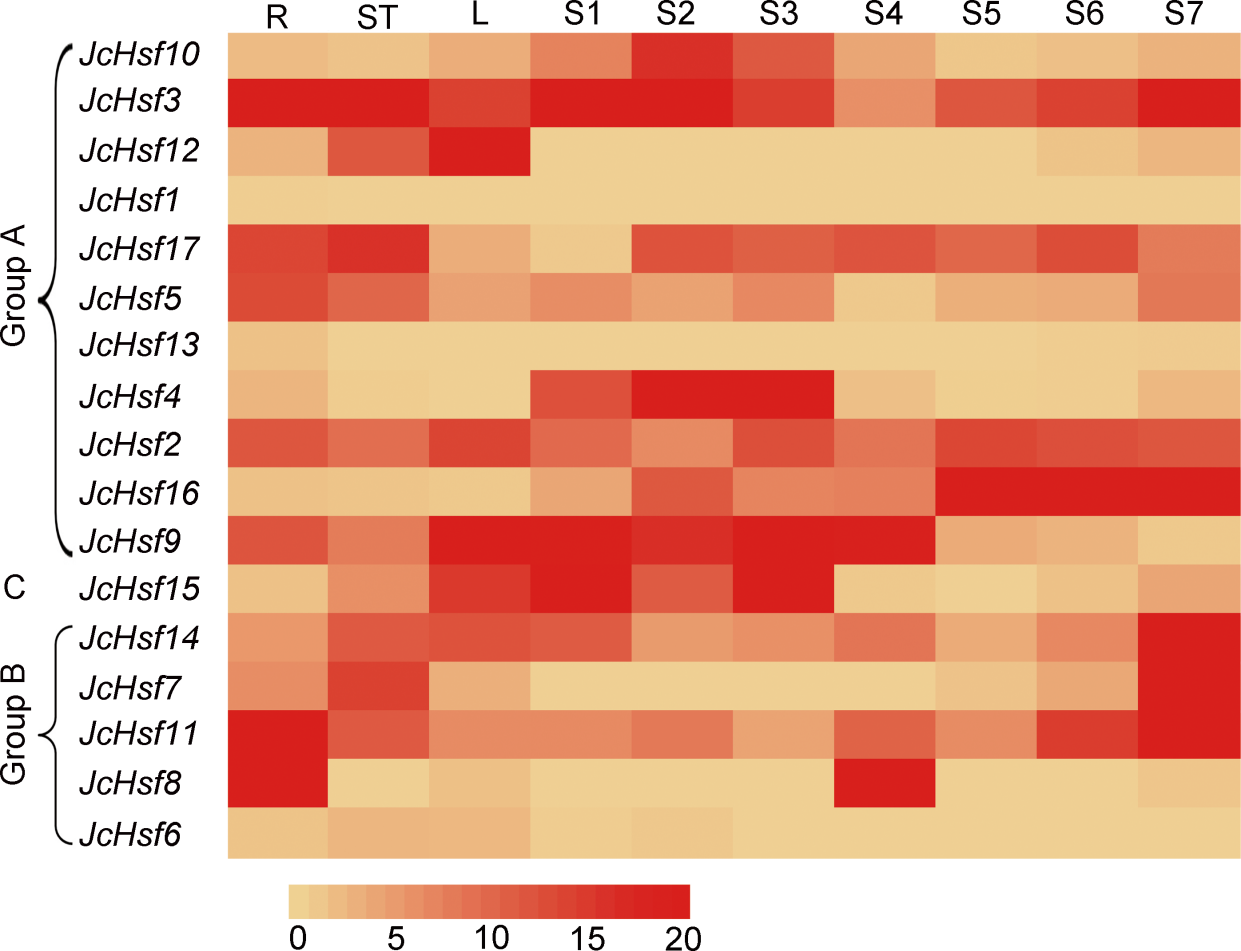
Expression analysis of the *JcHsf* genes in different tissues. R: roots, St: stem cortex, L: leaves S1–S7 correspond to seven different developing physic nut seeds: on 14, 19, 25, 29, 35, 41, and 45 days after pollination. The color scale represents signal values.

To gain insight into the function of *JcHsf* genes, their expression patterns in different tissues were analyzed. Results showed that *JcHsf1* was not expressed in all the tissues, *JcHsf6* and *JcHsf13* with low expression levels were shown in different tissues and developing seeds. Of the other fourteen *JcHsf* genes, four (*JcHsf3*, *5*, *11* and *8*), two (*JcHsf7*, *17*) and three (*JcHsf2*, *9* and *12*) showed the highest expression level in the root, stems and leaves, respectively. Four (*JcHsf4*, *9*, *10* and *15*) showed a much higher expression level in the seed early developing stage (S1–S4). Six (*JcHsf2*, *11*, *14* and *16*) showed much higher expression level in the seed late developing stage (S5–S7). Two (*JcHsf3* and *17*) showed sustained high expression throughout the seed development stages (Fig. 8).

### Expression profiles of *JcHsf* genes under drought and salt stress

**Figure 9:**
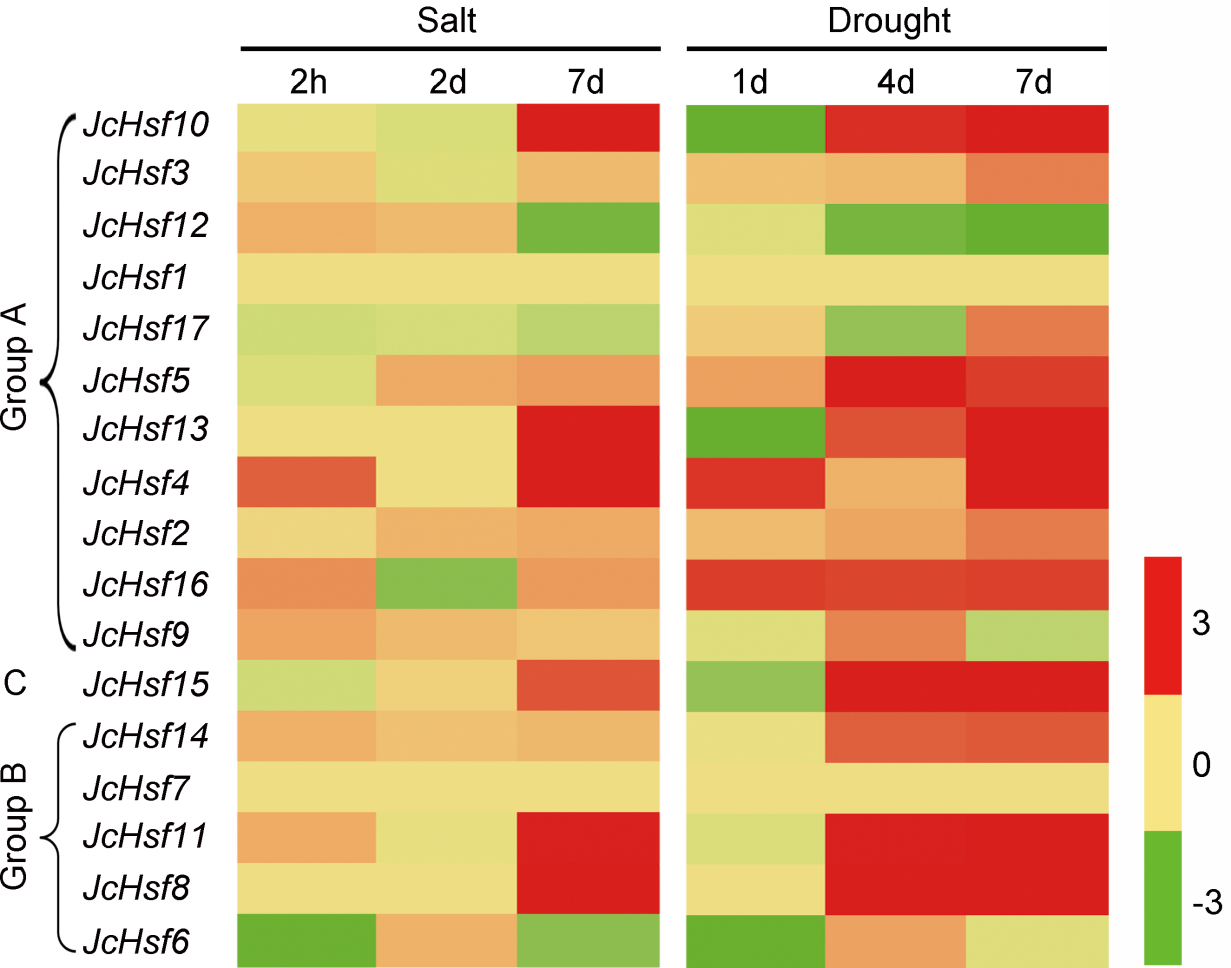
Expression analysis of *JcHsf* genes in leaves under salt and drought stress. Log_2_ based values (signal values for treatment/signal value for control) were used to create the heat map based on transcriptomic data for *Hsfs*. The color scale represents signal values.

In order to identify the expression pattern of *JcHsf* genes in response to salt and drought stress, the reported expression profile data of these genes in leaves 2 h, 2d and 7d under salt stress (Zhang et al., 2014) and 1d, 4d and 7d under drought stress (Zhang et al., 2015b) were analyzed (Fig. 9). The results showed that six genes (*JcHsf4*, *8*, *10*, *11*, *13* and *15*) were significantly up-regulated and two genes (*JcHsf* 6 and *12*) were down regulated in both salt and drought stress condition. In addition, the other six genes (*JcHsf2*, *3*, *5*, *14*, *16*, *17*) highly up-regulated in response to drought were also identified.

**Figure 10:**
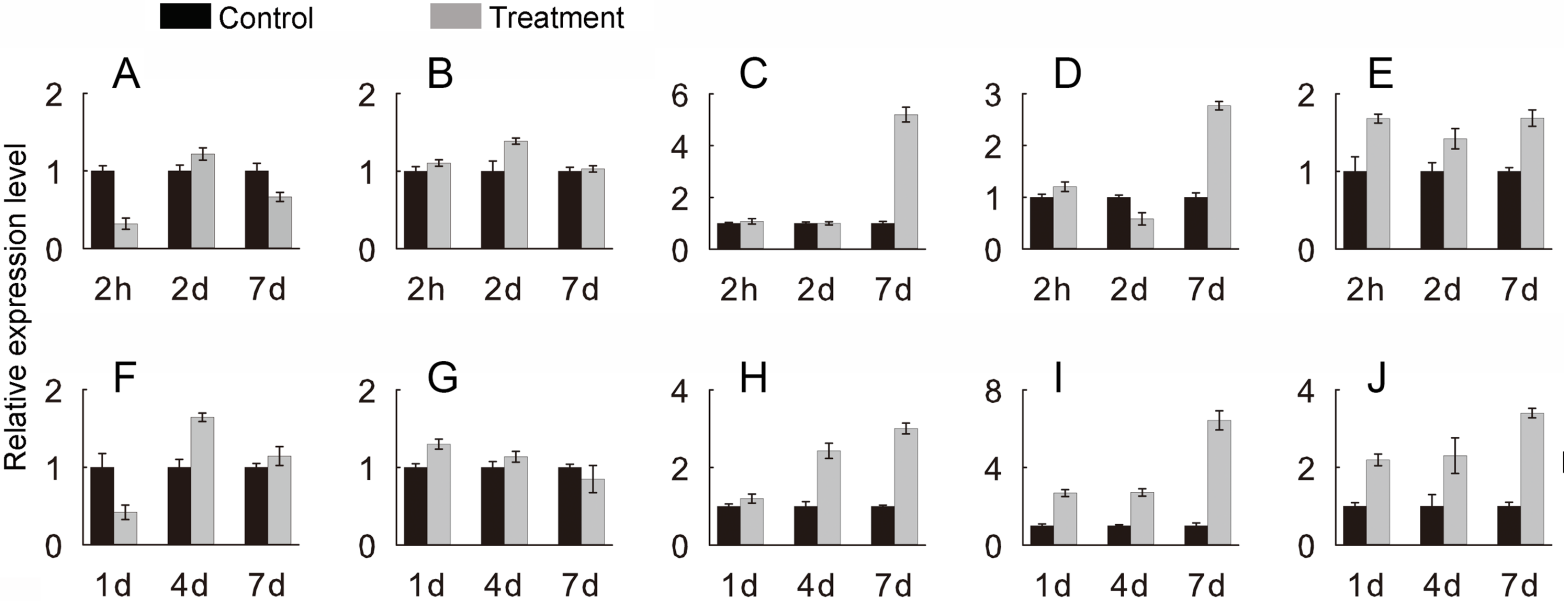
Expression level of selected *JcHsf* genes. qRT-PCR analysis of selected genes in leaves after salt and drought treatments at different time points. (A) *JcHsf6* under salt stress. (B) *JcHsf7* under salt stress. (C) *JcHsf8* under salt stress. (D) *JcHsf11* under salt stress. (E) *JcHsf14* under salt stress. (F) *JcHsf6* under drought stress. (G) *JcHsf7* under drought stress. (H) *JcHsf8* under drought stress. (I) *JcHsf11* under drought stress. (J) *JcHsf14* under drought stress. Relative expression was normalized with *JcActin* as the reference gene. Bars show standard deviations of the repeats. The experiment involved three biological replicates, each with two technological replicates.

To verify the *JcHsf* genes expression pattern from the RNA-seq data, the qRT-PCR were performed to detect the transcriptional level of group B *JcHsf* (*6*, *7*, *8*, *11* and *14*) in leaves under salt and drought stress (Fig. 10). The results were basically consistent with the changes in the expression of RNA-seq, indicating that the data of RNA-seq were generally accurate.

## Discussion

Physic nut has been emerged as a renewable resource for biodiesel production, which can be grown on the barren soil (King et al., 2009; Divakara et al., 2010). Until now, little is known about the response mechanism to abiotic stress in physic nut. More and more evidence suggests that Hsfs play central roles in plant developmental and response to abiotic stresses (Lin et al., 2018; Lohani et al., 2019). However, there have been no reports of any studies on the *Hsf* genes in physic nut, so it is necessary to investigate the new *Hsf* genes in physic nut. In this study, 17 *Hsf* genes were found in physic nut genome (Table 1) (Wu et al., 2015).

Phylogenetic tree suggested that, JcHsf proteins could be divided into three groups (Fig. 2), similar to *Arabidopsis* and other plants (Guo et al., 2008; Lin et al., 2011; Liu et al., 2016). The *Hsf* gene numbers of some specific subgroups in physic nut were different from *Arabidopsis*. For example, the number of subgroup A1, A6 and A7 in physic nut were less than that in *Arabidopsis*. One possible reason is that during the early stages of evolution, *JcHsf* genes have not yet experienced chromosome fragment replicate events (Wu et al., 2015). Another possible reason is that the *Arabidopsis* may acquire the *Hsf* genes while the physic nut lost it from their common ancestor (Wu et al., 2015).

Gene’s structure analyses indicated that all *Hsf* genes were found to contain only one intron (Fig. 3). Although physic nut *Hsf* genes shared same intron number, the intron length differed across the groups, which revealed the very highly conserved exon/intron splicing arrangement. Notably, *JcHsf4* and *JcHsf7* in the subgroup A1 has a long intron which made them different from other *JcHsf* gene members because of their larger size (9,741 bp and 7,519 bp respectively). In general, the gene structure of members in the same class has the similar domain or motif. In this study, although motif 3 and 6 were included in coiled coil structure, but motif 3 was only presented in group A and C, while motif 6 was just detected in group B (Fig. 6). These motifs were specific to some group, which are awaited for participating in group-specific function. Additionally, there are no AHA motifs required for transcriptional activity in the three members of group A (JcHsf3, JcHsf9 and JcHsf16). Previous studies showed that these proteins may gain function through binding to other menbers of group A to form hetero-oligomers (Guo et al., 2008).

Gene’s expression profiles are often related to their functions (Guo et al., 2008). In the present study, the expression profiles of each *JcHsf* gene in roots, stems, leaves and developing seeds were investigated (Jiang et al., 2012). Most *JcHsfs* were detected highly expressed in different tissues and developing seeds. It illustrated that *Hsf* genes are critical regulators involved in plant growth and development (Fig. 8). In addition, *JcHsf10* expression was highest in S2 stage of developing seeds (Fig. 8); its homolog *AtHsfA1b* was reported to regulate multiple developmental genes under heat stress in *Arabidopsis* (Albihlal et al., 2018). Therefore, it can be inferred that, at the early stage of developing seed; high level of *JcHsf10* may take part in regulating seed development of physic nuts under heat stress.

Studies on genome-wide expression analysis of different plants showed that the expression level of some *Hsf* genes changed in response to different abiotic stresses (Mittal et al., 2009; Guo et al., 2015; Dossa, Diouf & Cisse, 2016; Liu et al., 2016; Lohani et al., 2019). Consistent with this, 14 *JcHsf* genes out of 17 were up- or down-regulated under at least one stress condition (Fig. 9). Among these 14 genes, 11 genes showed at least 2 fold up- or down-regulated in at least one-time point in response to both salt and drought stimuli (Fig. 9), which is consisted with previous studies on *Hsfs* in various plant species (Chauhan et al., 2011; Li et al., 2014; Dossa, Diouf & Cisse, 2016). Overexpression *Arabidopsis HsfA1a* had a positive effect on tolerance to various stressors by acting the inducible heat shock protein expression (Qian et al., 2014). Its physic nut homolog *JcHsf3* was highly expressed in all tested tissues (Fig. 8) and up-regulated significantly at 7d in leaves after drought stress (Fig. 9), suggesting a role response to drought stress. *AtHsfA2* is essential for acquiring thermotolerance in *Arabidopsis* (Charng et al., 2007), and its homolog *JcHsf17* presented high level in roots, stems and after S2 stage of developing seeds (Fig. 8), and also up-regulated significantly at 7d in leaves after drought stress (Fig. 9). The result suggests that *JcHsf17* may play roles in both thermo and drought tolerance in physic nut. In addition, *AtHsfB1* and *AtHsfB2b*, took part in regulating the expression of defensin gene *Pdf1.2*, plays an important role in pathogen resistance (Kumar et al., 2009), and their homolog in physic nut were *JcHsf11* and *JcHsf14*, respectively. *JcHsf11* and *JcHsf14*, both classified into group B, presented similar expression pattern under normal and abiotic condition (Figs. 8 and 9). It indicated that *JcHsf11* and *JcHsf14* may involve in pathogen resistance in physic nut. In brief, we speculate that *JcHsf* genes may participate in many aspects of the developmental process in physic nut, and their roles deserve further study.

## Conclusions

In summary, 17 *JcHsf* genes were identified from the physic nut genome, and gene information provided, including chromosomal localization, phylogenetic and structure analysis. Most *JcHsf* genes showed a differential expression pattern, indicating the great significance of *JcHsf* in plant development and stress response. Although the exact function of the *JcHsf* genes cannot still be indicated, this study lays a foundation for further study on its potential role in regulating developmental process and stress response in physic nut.

##  Supplemental Information

10.7717/peerj.8467/supp-1 Table S1 Primers used in this study

10.7717/peerj.8467/supp-2 Table S2 List of plant Hsfs accession numbers

10.7717/peerj.8467/supp-3 Table S3 Motif sequences identified using MEME tools in physic nut HSFs

## Additional Information and Declarations

### Data Availability

The following information was supplied regarding data availability: Data is available at NCBI SRA: PRJNA257901, PRJNA244896.
