# Supplementary material for: Genome-wide analysis and expression profiling of the heat shock transcription factor gene family in Physic Nut (Jatropha curcas L.)
Source: PeerJ. 2020 Feb 5;8:e8467. doi: 10.7717/peerj.8467 (PMC7007736; doi:10.7717/peerj.8467)
Supplement: Table S1 [file peerj-08-8467-s001.docx]

Table S1 Primers used in this study.

| Primer Name | Primer sequences(5’>3’) |
| --- | --- |
| JcHsf6-Forward | TCTCTAGCTTCATTCGCCAACT |
| JcHsf6-Reverse | TCCTCGTTATCTCCACCAGCAT |
| JcHsf7- Forward | TGGTGTAGCGATTGGCATCAAG |
| JcHsf7- Reverse | TCACTGCATTGGCTCCTGGTT |
| JcHsf8- Forward | CGGTGGGAGTTCTGCAATGACA |
| JcHsf8- Reverse | ATCGGATTCTTGCGGTGTGGTT |
| JcHsf11- Forward | TAGCTGCCTCGCCATCGAACTC |
| JcHsf11- Reverse | ATTGTGCCGTTGCCGTCATTGT |
| JcHsf14- Forward | GCAACAACGGCTACAGTGACC |
| JcHsf14- Reverse  JcActin- Forward  JcActin- Reverse | ACCTGCTCATCTCCCGAAGTC  TAATGGTCCCTCTGGATGTG  AGAAAAGAAAAGAAAAAAGCAGC |
