# Supplementary material for: Genome-wide analysis and expression profiling of the heat shock transcription factor gene family in Physic Nut (Jatropha curcas L.)
Source: PeerJ. 2020 Feb 5;8:e8467. doi: 10.7717/peerj.8467 (PMC7007736; doi:10.7717/peerj.8467)
Supplement: Table S2 [file peerj-08-8467-s002.docx]

Table S2 List of plant Hsfs accession numbers.

| AtHsfs | Arabidopsis (21) | OsHsfs | Rice (25) | VvHsfs | Grape (17) | JcHsfs | Physic nut (17) |
| --- | --- | --- | --- | --- | --- | --- | --- |
| HsfA1a | At4g17750 | OsHsf1 | LOC_Os01g39020 | VvHSF1 | GSVIVP00001119001 | JcHsf1 | JCGZ_02229 |
| HsfA1 b | At5g16820 | OsHsf2 | LOC_Os01g43590 | VvHSF2 | GSVIVP00007367001 | JcHsf2 | JCGZ_01081 |
| HsfA1 c | At1g32330 | OsHsf3 | LOC_Os01g53220 | VvHSF3 | GSVIVP00013075001 | JcHsf3 | JCGZ_17108 |
| HsfA 1d | At3g02990 | OsHsf4 | LOC_Os01g54550 | VvHSF4 | GSVIVP00013429001 | JcHsf4 | JCGZ_04789 |
| HsfA2a | At2g26150 | OsHsf5 | LOC_Os02g13800 | VvHSF5 | GSVIVP00014836001 | JcHsf5 | JCGZ_21617 |
| HsfA3a | At5g03720 | OsHsf6 | LOC_Os02g29340 | VvHSF6 | GSVIVP00015710001 | JcHsf6 | JCGZ_21433 |
| HsfA4a | At4g18880 | OsHsf7 | LOC_Os02g32590 | VvHSF7 | GSVIVP00016537001 | JcHsf7 | JCGZ_26137 |
| HsfA4b | At5g45710 | OsHsf8 | LOC_Os03g06630 | VvHSF8 | GSVIVP00016746001 | JcHsf8 | JCGZ_21103 |
| HsfA5a | At4g13980 | OsHsf9 | LOC_Os03g12370 | VvHSF9 | GSVIVP00017248001 | JcHsf9 | JCGZ_06744 |
| HsfA6a | At5g43840 | OsHsf10 | LOC_Os03g25120 | VvHSF10 | GSVIVP00018812001 | JcHsf10 | JCGZ_02539 |
| HsfA6b | At3g22830 | OsHsf11 | LOC_Os03g53340 | VvHSF11 | GSVIVP00019488001 | JcHsf11 | JCGZ_12391 |
| HsfA7a | At3g51910 | OsHsf12 | LOC_Os03g58160 | VvHSF12 | GSVIVP00020055001 | JcHsf12 | JCGZ_21870 |
| HsfA7b | At3g63350 | OsHsf13 | LOC_Os03g63750 | VvHSF13 | GSVIVP00021673001 | JcHsf13 | JCGZ_03936 |
| HsfA8a | At1g67970 | OsHsf14 | LOC_Os04g48030 | VvHSF14 | GSVIVP00023801001 | JcHsf14 | JCGZ_20639 |
| HsfA9a | At5g54070 | OsHsf15 | LOC_Os05g45410 | VvHSF15 | GSVIVP00030611001 | JcHsf15 | JCGZ_07238 |
| HsfB1a | At4g36990 | OsHsf16 | LOC_Os06g35960 | VvHSF16 | GSVIVP00032192001 | JcHsf16 | JCGZ_07430 |
| HsfB2a | At5g62020 | OsHsf17 | LOC_Os06g36930 | VvHSF17 | GSVIVP00035821001 | JcHsf17 | JCGZ_07655 |
| HsfB2b | At4g11660 | OsHsf18 | LOC_Os07g08140 |  |  |  |  |
| HsfB3a | At2g41690 | OsHsf19 | LOC_Os07g44690 |  |  |  |  |
| HsfB4a | At1g46264 | OsHsf20 | LOC_Os08g36700 |  |  |  |  |
| HsfC1a | At3g24520 | OsHsf21 | LOC_Os08g43334 |  |  |  |  |
|  |  | OsHsf22 | LOC_Os09g28200 |  |  |  |  |
|  |  | OsHsf23 | LOC_Os09g28354 |  |  |  |  |
|  |  | OsHsf24 | LOC_Os09g35790 |  |  |  |  |
|  |  | OsHsf25 | LOC_Os10g28340 |  |  |  |  |

Note: Web pages for blast searches and gene identification are: <http://plntfdb.bio.uni-potsdam.de/v3.0/> : Rice, Arabidopsis, Grape. <https://www.ncbi.nlm.nih.gov/> : physic nut.
