# Supplementary material for: Genome-wide analysis and expression profiling of the heat shock transcription factor gene family in Physic Nut (Jatropha curcas L.)
Source: PeerJ. 2020 Feb 5;8:e8467. doi: 10.7717/peerj.8467 (PMC7007736; doi:10.7717/peerj.8467)
Supplement: Table S3 [file peerj-08-8467-s003.docx]

Table S3 Motif sequences identified using MEME tools in physic nut HSFs.

| Motif | Multilevel consensus sequence | Function |
| --- | --- | --- |
| 1 | FIVWDPPEFARDLLPKYFKHNNFSSFVRQLNTYGFRKVDPDRWEFANEGF | DBD |
| 2 | PPPFLTKTYEMVDDPSTDSIVSWSESGNS | DBD |
| 3 | LMQELVRLRQQQQNTENQLQALEQRLQSMEQRQQQMMSFLAKAMQNPGFL | [coiled coil](javascript:domWin(1)) |
| 4 | LRGQKHLLKNIRRRKPSQSH | DBD |
| 5 | QQQSLGACVEVGRFGLEGEVERLKRDRNV | Unknown |
| 6 | LLEENERLKKENLQLSSELTQMKALCNELLALLANYA | [coiled coil](javascript:domWin(1)) |
| 7 | VNDVFWEQFLTEDPGSSNTDE | AHA |
| 8 | AIGKKRRL | NLS |
| 9 | WWNAQNMNQLTEQMGLLASA | NES |
| 10 | PQPMEGLHETG | Unknown |

Note : Numbers in the first column indicate the motifs represented in Figure 6 and 7.
